# Nonlinear temperature sensitivity of enzyme kinetics explains canceling effect—a case study on loamy haplic Luvisol

**DOI:** 10.3389/fmicb.2015.01126

**Published:** 2015-10-14

**Authors:** Bahar S. Razavi, Evgenia Blagodatskaya, Yakov Kuzyakov

**Affiliations:** ^1^Department of Agricultural Soil Science, University of GöttingenGöttingen, Germany; ^2^Department of Soil Science of Temperate Ecosystems, University of GöttingenGöttingen, Germany; ^3^Institute of Physicochemical and Biological Problem in Soil Science, Russian Academy of SciencePushchino, Russia

**Keywords:** carbon cycle, Michaelis-Menten kinetics, Arrhenius function, soil enzymes, temperature sensitivity, canceling effect, activation energy

## Abstract

The temperature sensitivity of enzymes responsible for organic matter decomposition in soil is crucial for predicting the effects of global warming on the carbon cycle and sequestration. We tested the hypothesis that differences in temperature sensitivity of enzyme kinetic parameters *V*_*max*_ and *K*_*m*_ will lead to a canceling effect: strong reduction of temperature response of catalytic reactions. Short-term temperature response of *V*_*max*_ and *K*_*m*_ of three hydrolytic enzymes responsible for decomposition of cellulose (β-glucosidase, cellobiohydrolase) and hemicelluloses (xylanase) were analyzed *in situ* from 0 to 40°C. The apparent activation energy varied between enzymes from 20.7 to 35.2 kJ mol^−1^ corresponding to the *Q*_10_ values of the enzyme activities of 1.4–1.9 (with *V*_*max*_**-***Q*_10_ 1.0–2.5 and *K*_*m*_**-***Q*_10_ 0.94–2.3). Temperature response of all tested enzymes fitted well to the Arrhenius equation. Despite that, the fitting of Arrhenius model revealed the non-linear increase of two cellulolytic enzymes activities with two distinct thresholds at 10–15°C and 25–30°C, which were less pronounced for xylanase. The nonlinearity between 10 and 15°C was explained by 30–80% increase in *V*_*max*_. At 25–30°C, however, the abrupt decrease of enzyme-substrate affinity was responsible for non-linear increase of enzyme activities. Our study is the first demonstrating nonlinear response of *V*_*max*_ and *K*_*m*_ to temperature causing canceling effect, which was most strongly pronounced at low substrate concentrations and at temperatures above 15°C. Under cold climate, however, the regulation of hydrolytic activity by canceling in response to warming is negligible because canceling was never observed below 10°C. The canceling, therefore, can be considered as natural mechanism reducing the effects of global warming on decomposition of soil organics at moderate temperatures. The non-linearity of enzyme responses to warming and the respective thresholds should therefore be investigated for other enzymes, and incorporated into Earth system models to improve the predictions at regional and global levels.

## Introduction

The temperature sensitivity of soil organic matter (SOM) decomposition has attracted significant interest because of its importance in the global carbon cycle (C) and potential feedbacks to global warming (Schimel, 1995; Davidson and Janssens, 2006; De Graaff et al., 2014). Temperature sensitivity of reaction rates (e.g., SOM decomposition) is commonly described by the Arrhenius equation. This is based on the energy required to initiate the reaction, termed the activation energy (*E*_*a*_), (Von Lützow and Kögel-Knabner, 2009). For chemical reactions, the Arrhenius equation predicts exponentially increasing reaction rates with increasing temperature, assuming constant values of activation energy (Kirschbaum, 2000; Craine et al., 2010; Craine and Gelderman, 2011). In nature, however, the decomposition of SOM is mediated by extracellular enzymes, produced by microorganisms (Allison et al., 2010; Glanville et al., 2012; Zimmermann and Bird, 2012; Van Gestel et al., 2013). Therefore, deviations from Arrhenius behavior can occur as a consequence of temperature sensitivity of enzyme systems, through enzyme denaturation and proteolysis, for example (Bennett et al., 2008; Maire et al., 2013; Goyal et al., 2014) or by temperature-accelerated desorption of immobilized enzymes (Nannipieri et al., 1996; Nielsen et al., 2006). Besides that, changes in the temperature dependency of microbial communities may cause expression of various set of isoenzymes (i.e., an enzyme with the same function but a different structure) or changes in enzyme conformation (Bradford, 2013). This, in turn, affects apparent activation energy and temperature sensitivity of enzyme-mediated reactions *in situ* in soil.

Besides enzyme properties, the rate of enzyme-mediated reactions is dependent on substrate amount, which can strongly vary in time and space considering heterogeneity of soil microhabitats (Cheng et al., 1996; Ekschmitt et al., 2005; Ruamps et al., 2013). Therefore, not only the Arrhenius equation, but the relationship between rate of enzyme-mediated reaction and substrate needs to be considered in the context of temperature sensitivity of SOM decomposition. The rate of catalytic reactions is a saturating function of substrate concentration and is described by the Michaelis-Menten relationship (Michaelis and Menten, 1913). Both parameters of the Michaelis-Menten equation—maximum enzyme activity (*V*_*max*_) and the half-saturation constant (*K*_*m*_), which is an intrinsic feature of an enzyme system reflecting the affinity of the enzyme for the substrate (Khalili et al., 2011; Bradford, 2013)—are temperature sensitive (Davidson and Janssens, 2006; Davidson et al., 2006; Stone et al., 2012). As both *V*_*max*_ and *K*_*m*_ values (respectively, in the numerator and denominator of the Michaelis-Menten equation) usually increase with temperature (Stone et al., 2012), a canceling effect (absence or strong reduction of response of the enzyme to temperature) can occur for the resulting enzymatic reaction rate (Berry and Raison, 1981; Atkin and Tjoelker, 2003; Davidson et al., 2006). The canceling effect may be pronounced when the substrate concentration is lower than or close to *K*_*m*_ and if both *K*_*m*_ and *V*_*max*_ have similar temperature sensitivities (Larionova et al., 2007; Gershenson et al., 2009). Ecological and evolutionary processes in microbial communities could also reduce the temperature sensitivity of enzymes by reducing *V*_*max*_ and increasing *K*_*m*_ (Hochachka and Somero, 2002). It should be noted that response of pure and isolated enzyme are strongly different compared to the soil enzyme, for instance, the catalytic properties of the soil enzymes (e.g., substrate affinity) are much higher compared to the bacterial and fungal enzymes originated from pure cultures (Skálová et al., 2005; Tischer et al., 2015).

Relative temperature responses are commonly compared by a *Q*_10_ index—the change of a reaction rate with a temperature increase of 10°C (Birgander et al., 2013). *Q*_10_ values of 2–3 are commonly assumed for respiration rates in the temperature interval 10–20°C (Davidson and Janssens, 2006). However, enzyme activities have been found to be less temperature sensitive than compared to respiration, with *Q*_10_ values < 2 (Browman and Tabatabai, 1978; Tabatabai, 1994; Koch et al., 2007), and lower *Q*_10_ values are often observed at higher temperatures (Tjoelker et al., 2001; Xu and Qi, 2001). Recently, a cross-latitudinal study for enzymes involved in the C cycle demonstrated *V*_*max*_**-***Q*_10_ values ranging from 1.5 to 2.3 and *K*_*m*_**-***Q*_10_ values ranging from 0.90 to 1.9 (German et al., 2012). When *V*_*max*_ and *K*_*m*_ cancel each other out (i.e., the *Q*_10_ of catalytic reaction ~1), decomposition is restricted by temperature sensitivity of a bottle-neck process that produces available substrate, e.g., by decomposition of recalcitrant or stabilized SOM (Ågren and Wetterstedt, 2007). The canceling effect is usually more pronounced when substrate concentration is lower or close to *K*_*m*_ and if both *K*_*m*_ and *V*_*max*_ have similar temperature sensitivities (Larionova et al., 2007; Gershenson et al., 2009). Accordingly, the canceling effect can be an important factor controlling the “actual” temperature sensitivity of organics decomposition in soils (Von Lützow and Kögel-Knabner, 2009). Despite theoretical predictions (Davidson and Janssens, 2006), there is still a lack of experimental data on the occurrence of canceling as dependent on temperature range, substrate amount and enzyme.

This study was designed to test how the canceling effect of three enzymes involved in the C cycle changes with temperature. We hypothesized that the temperature sensitivity of *V*_*max*_ and *K*_*m*_ differ both: (1) in the specific *Q*_10_, and (2) in the temperature ranges with maximal changes of enzyme activity and substrate affinity. The consequence of this hypothesis is that the increase in the rate of enzymatic reaction with temperature is not continuous, but may have some thresholds with a much stronger response. Using the modified Arrhenius equation we tested the response of enzyme systems to temperature, e.g., thermal denaturation or changes in enzyme affinity to substrate.

The modern global change models simulate the responses of soil C pools to about 4.8°C increase over the next 100 years (IPCC, 2007; Wieder et al., 2013). Because the main C input in soil is ongoing by above and belowground litter (Hasibeder et al., 2014), it is crucial to understand how enzymes involved in litter decomposition will respond to warming to better predict the links between C input, SOM formation and global warming (German et al., 2011). Thus, soil (haplic Luvisol) was incubated over a temperature range of 0–40°C (with 5° steps) during 5 days to determine the activities of three enzymes: β-glucosidase and cellobiohydrolase, which are responsible for degrading cellulose (Mganga et al., 2015; Wang et al., 2015), and xylanase, which degrades xylooligosaccharides (short xylene chain) into xylose and is thus responsible for breaking down hemicelluloses (Chen et al., 2012).

## Material and methods

### Soil

Soil samples were taken from the top 10 cm of the Ap horizon of an arable loamy haplic Luvisol located on a terrace plain of the Leine River north-west of the city of Göttingen (Holtensen, Germany) with mean annual temperature 5–15°C. The properties of the soil were: pH 6.5; 5.8% sand, 87.2 % silt, 5.8 % clay; 12.6 g kg^−1^ C, 1.3 g kg^−1^ N, 1.4 g cm^−3^ bulk density, and 30 % water field capacity. The samples were kept cold (~4°C) during transportation to the laboratory. Then the samples were frozen at −20°C until pre-incubation.

### Temperature incubation experiment

Three enzymes involved in the C cycle were investigated over a temperature range of 0, 5, 10, 15, 20, 25, 30, 35, and 40°C. Nine climate chambers (SBS C120) were used to regulate the temperature with a precision of < 1°C. At each temperature four replicates were incubated. The frozen samples were thawed at 4°C for 1 day and then pre-incubated at 20°C for 14 days before the start of 5 days incubation. Because freezing may affect enzymatic activities (Lee et al., 2007; Stone et al., 2012), all samples were frozen similarly, and they were pre-conditioned after thawing. We therefore assume that this pretreatment corresponded to snow thaw in spring and that the freezing effect was negligible after the pre-incubation.

### Enzyme assays

The kinetics of enzyme activities was assayed using fluorogenically labeled substrates. Three types of fluorogenic substrates based on 4-methylumbelliferone (MUF) were used to assess enzymatic activities; 4-methylumbelliferyl-β-D-glucoside (MUF-G) to detect β-glucosidase activity; 4-methylumbelliferyl-β-D-cellobioside (MUF-C) to detect cellobiohydrolase activity; and 4-methylumbelliferyl-β-D-xylopyranoside (MUF-X) to detect xylanase activity. All substrates and chemicals were purchased from Sigma (Germany).

MUF-substrates were dissolved in 2-methoxyethanol (Hoppe, 1983). Pre-dissolved MUF-substrates were further diluted with sterile universal buffer [MES (C_6_H_13_NO_4_SNa_0.5_)] to obtain the desired concentrations; we determined enzyme activities in a range of substrate concentrations from low to high (0, 10, 20, 30, 40, 50, 100, 200 μmol g^−1^soil). Saturation concentrations of fluorogenic substrates were determined in preliminary experiments. Suspensions of 0.5 g soil (dry weight equivalent) with 50 mL water were prepared separately for each of 4 incubated replicates using low-energy sonication (40 J s^−1^ output energy) for 2 min (Stemmer et al., 1998; Koch et al., 2007). Fifty microliters of soil suspension was added to 50 μL buffer (pH:6.5) and 100 μL of each substrate solution in a 96-well microplate (Puregrade, Germany). The empty microplates as well as working solutions were pre-incubated at the exact temperature of the assay before the experiment started. The calibration solutions were prepared using soil suspension (50 μL) and MUF to obtain a series of concentrations 0–1.2 mM (Ali et al., 2015). Fluorescence was measured in microplates at an excitation wavelength of 355 nm and an emission wavelength of 460 nm, slit width of 25 nm, with a Victor3 1420-050 multi label counter (Perkin Elmer, USA). Activity of each enzyme was determined in each soil sample at exact temperature during 3 h. No enzyme assay took longer than 2 min. After each measurement (i.e., after 30 min, 1 h, 2 h, and 3 h) the microplates were promptly returned to the climate chambers.

Enzyme activities were expressed as MUF release in nmol per g dry soil per hour (nmol g^−1^ h^−1^). In addition for all four incubation replicates, the assay of each enzyme at each substrate concentration was performed in three analytical replicates (12 wells in the microplate). Due to high-temperature effects, the chemical reactions of the substrates are considerable. As thermal hydrolysis of MUF-phosphate could occur at temperatures of 40–65°C (Spohn and Kuzyakov, 2013), we checked possible temperature effects on the chemical decomposition of the three MUF-substrates, but no significant effects were detected in the range 0–40°C.

### Enzyme kinetics, temperature sensitivity, and statistical analyses

The Michaelis-Menten equation used to determine parameters of enzyme activity (V),

(1) $$\begin{array}{l} V=\frac{V_{\max}\times \left[S\right]}{K_{m}+\left[S\right]} \end{array}$$

Where, *V*_*max*_ is the maximal rate of enzymatic activity at a given temperature; *K*_*m*_ is the half saturation constant, or the substrate concentration at ½*V*_*max*_, and [*S*] is the concentration of the substrate (Michaelis and Menten, 1913; Segel, 1975). All parameters were modeled with the non-linear regression routine of STATISTICA. The *t*-test (pairwise differences) was applied to distinguish the significant differences for each neighboring pair of independent variables (mean values of *V*_*max*_ at 0, 5, 10, 15, 20, 25, 30, 35, 40°C), then the temperatures with insignificant differences in V_*max*_ were selected in one temperature-range group (Boone et al., 1998; Melillo et al., 2002). Thus, based on the *t*-test, the enzyme activities were not significantly different within each categorical group, but they were significantly different from the other groups. Thereafter, One-way analysis of variance (ANOVA) at a probability level of *p* < 0.05, was applied to distinguish the significant differences in *K*_*m*_ values between categorical temperature-range groups (Table S1). Homogeneity of variance and normality of the values was tested by the Leven's test and Shapiro-wilk's *W*-test.

We used the conventional *Q*_10_ function (2) to examine variation in temperature sensitivity and express temperature responses of each enzyme kinetic parameter (i.e., *K*_*m*_ or *V*_*max*_).

(2) $$Q10=\left(\frac{{}^{R}(T\text{ + }10^{{}^{\circ}}C)}{{}^{R}(T)}\right)$$

where, *R* is the rate of a process or a value of kinetic parameters and *T* is temperature. For unification and comparison of canceling effects at 5and 10°C increment, we used function (3):

(3) $$\begin{array}{l} Q10={\left(\frac{R\left(T_{2}\right)}{R\left(T_{1}\right)}\right)}^{\frac{10}{\left(T_{2}-T_{1}\right)}} \end{array}$$

where, *R(T*_1_*)* and *R(T*_2_*)* are the rates of a process or reaction at two temperatures (Kirschbaum, 1995; Karhu et al., 2010, 2014; Khalili et al., 2011).

The activation energy was calculated according to the classical Arrhenius equation (Equation 4):

(4) $$\begin{array}{r} k=A\;exp\left(-E_{a}∕RT\right) \end{array}$$

where, *k* is the reaction rate constant; *A* is the frequency of molecular collisions; *E*_*a*_ is the required activation energy in Joules per mole; *R* is the gas constant (8.314 J mol^−1^ K^−1^) and *T* is the temperature in Kelvin. In addition to the classical Arrhenius equation (Equation 4), the modified Arrhenius equation (Equation 5) was applied to estimate possible effects of enzyme denaturation on apparent *E*_*a*_ that contributed to enzymes reaction rate (Flickinger and Drew, 1999).

(5) $$\begin{array}{r} \upsilon =\frac{A\;exp\left(-E_{a}∕RT\right)}{1+exp\left(\Delta S^{{}^{\circ}}∕R-\Delta H^{{}^{\circ}}∕RT\right)} \end{array}$$

Here, Δ*S*°and Δ*H*° are the entropy and enthalpy of denaturation, respectively, and υ is the observed rate of biological processes. Activity of β-glucosidase demonstrated similar temperature sensitivity to other enzymes (see Results) but the strongest pattern of both grouping for *V*_*max*_ and of non-linearity in temperature response for *K*_*m*_. We assumed, therefore, that any effect of thermal denaturation would be strongest for β-glucosidase as compared with the other tested enzymes. Hence, we applied the modified Arrhenius equation (Equation 7) to β-glucosidase activity, assuming intercept and absolute values of Δ*S*° and Δ*H*° determined in previous studies, using differential scanning calorimetry: Δ*S*° = 86 J mol^−1^ K^−1^ and Δ*H*° = 108 kJ mol^−1^, (Zoldák et al., 2004; Goyal et al., 2014).

The parameters A and *E*_*a*_ of Equations (4, 5) were fitted by the Marquardt minimization method using ModelMaker version 3.0.3 software (ModelMaker, 1997). The fitting quality and model efficiency were compared by the coefficient of determination (*R*^2^)

## Results

### Enzyme responses to temperature

**Figure 1:**
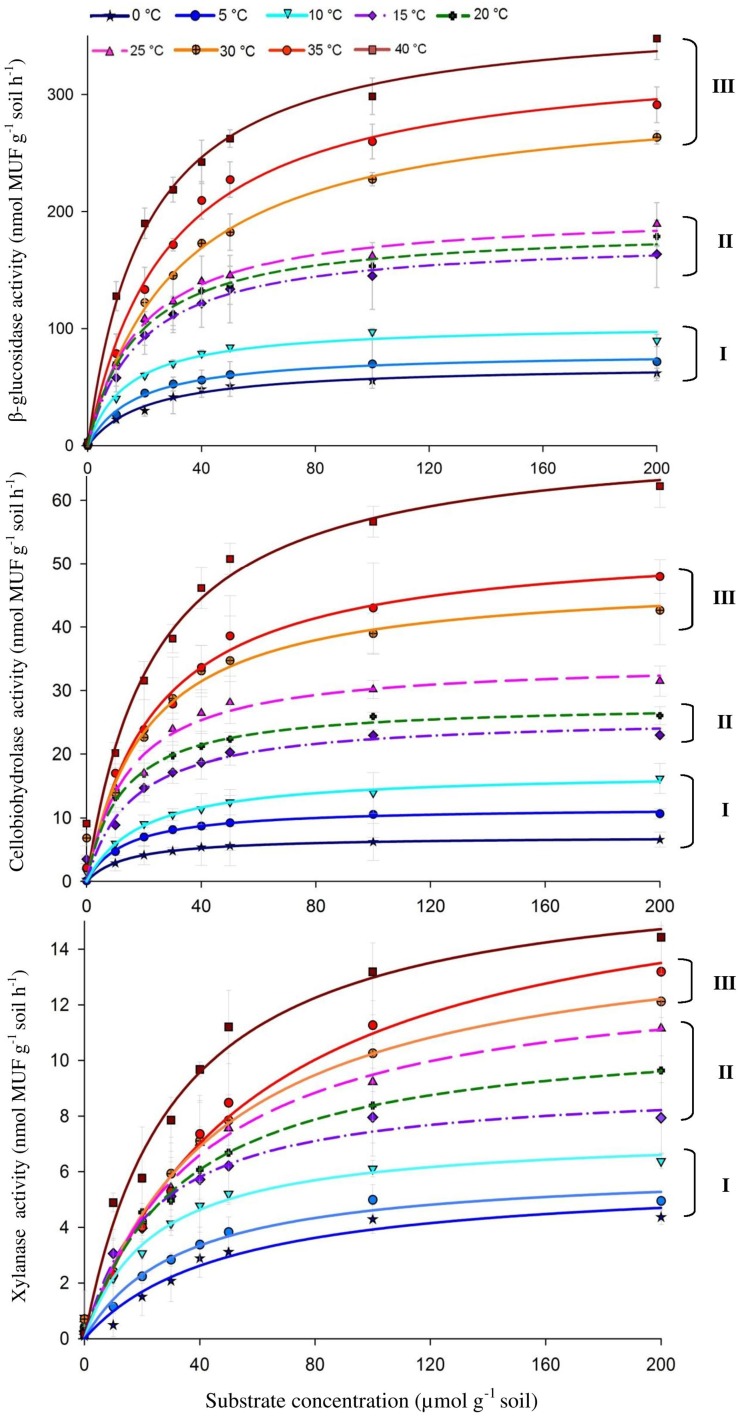
**Michaelis-Menten kinetics of β-glucosidase (top), Cellobiohydrolase (middle), and Xylanase (bottom) as a response to temperature increasing from 0 to 40°C with 5° steps**. Values are means of four replications (± SE). Curves present fitting of Michaelis-Menten kinetics by non-linear regression. The fitted *V*_*max*_ and *K*_*m*_ values are presented in Table 1. Grouping (I–III) was formed using the *t*-test based on significant differences between neighboring *V*_*max*_ values.

The activities of the three enzymes responded positively to increasing temperature with widest range of activity for β-glucosidase (from 69.9 to 362.8) and the narrowest one for xylanase (from 9.2 to 14.5) (Figure 1). The *Q*_10_ values for reaction rates were always >1, with the average range of 1.4–1.9. The Michaelis-Menten kinetics (enzyme activity as a function of substrate concentration) demonstrated stepwise increases in response to temperature across the whole range of substrate concentrations, i.e., 10–200 μmol g^−1^ (Figure 1). The temperature sensitivity of *V*_*max*_ and *K*_*m*_ decreased with increasing temperature and varied between enzymes, corresponding to *V*_*max*_**-***Q*_10_ values of 1.0–2.5 and *K*_*m*_**-***Q*_10_ values of 0.94–2.3 (Table 1).

**Table 1:** **The ***Q***_10_ values (± SE of four replicates) of ***V***_***max***_ and ***K***_***m***_ of three hydrolytic enzymes measured at nine temperatures**.

**Enzyme**	**Temperature °C**	***V_*max*_* (nmol h^−1^g^−1^)**	***K_*m*_* (μmol g^−1^soil)**	**Temperature range °C**	***Q*_10_*^*Vmax*^***	***Q*_10_*^*Km*^***
Xylanase	0	9.17 ± 0.51	19.38 ± 7.59	0–10	1.00 ± 0.06	1.09 ± 0.52
	5	6.17 ± 0.18	23.70 ± 2.76	5–15	1.97 ± 0.08	1.01 ± 0.16
	10	9.15 ± 0.14	21.16 ± 1.10	10–20	1.51 ± 0.24	1.46 ± 0.08
	15	12.14 ± 0.25	23.84 ± 2.62	15–25	1.05 ± 0.06	1.09 ± 0.21
	20	13.78 ± 2.17	30.82 ± 0.65	20–30	1.03 ± 0.20	1.59 ± 0.09
	25	12.75 ± 0.65	25.90 ± 4.14	25–35	1.22 ± 0.08	2.32 ± 0.39
	30	14.24 ± 0.34	49.04 ± 2.40	30–40	1.02 ± 0.03	1.15 ± 0.06
	35	15.55 ± 0.38	60.06 ± 2.94			
	40	14.47 ± 0.12	28.07 ± 0.24			
Cellobiohydrolase	0	9.12 ± 0.06	12.22 ± 0.48	0–10	2.32 ± 0.07	1.00 ± 0.16
	5	11.72 ± 0.08	13.16 ± 0.38	5–15	2.50 ± 0.08	1.04 ± 0.15
	10	18.24 ± 0.66	12.26 ± 1.86	10–20	1.51 ± 0.05	1.12 ± 0.18
	15	25.90 ± 0.90	13.66 ± 1.90	15–25	1.20 ± 0.06	1.02 ± 0.17
	20	28.70 ± 0.49	13.74 ± 1.68	20–30	1.50 ± 0.06	2.29 ± 0.24
	25	34.22 ± 0.79	13.90 ± 1.20	25–35	1.60 ± 0.05	2.01 ± 0.23
	30	49.19 ± 1.80	31.48 ± 2.64	30–40	1.51 ± 0.08	0.94 ± 0.16
	35	53.82 ± 1.20	27.96 ± 2.20			
	40	70.62 ± 2.16	29.46 ± 2.24			
β-glucosidase	0	69.99 ± 1.30	10.70 ± 1.20	0–10	1.50 ± 0.06	1.30 ± 0.17
	5	80.11 ± 0.91	16.86 ± 0.68	5–15	2.30 ± 0.07	1.00 ± 0.06
	10	101.89 ± 1.66	13.40 ± 1.10	10–20	1.96 ± 0.24	1.20 ± 0.11
	15	180.71 ± 2.03	17.76 ± 0.70	15–25	1.26 ± 0.06	1.10 ± 0.05
	20	199.90 ± 2.17	16.46 ± 0.46	20–30	1.61 ± 0.16	2.31 ± 0.15
	25	239.50 ± 1.50	18.40 ± 1.23	25–35	1.45 ± 0.08	1.60 ± 0.06
	30	301.96 ± 6.01	37.48 ± 1.84	30–40	1.10 ± 0.03	1.01 ± 0.06
	35	329.04 ± 3.64	29.92 ± 0.82			
	40	362.80 ± 6.70	39.30 ± 1.22			

*The temperature sensitivity of V_max_ and K_m_ decreased with increasing temperature and varied between enzymes. The temperature range column shows the ranges which were used to calculate V_max_**-**Q_10_ and K_m_**-**Q_10_ values*.

**Figure 2:**
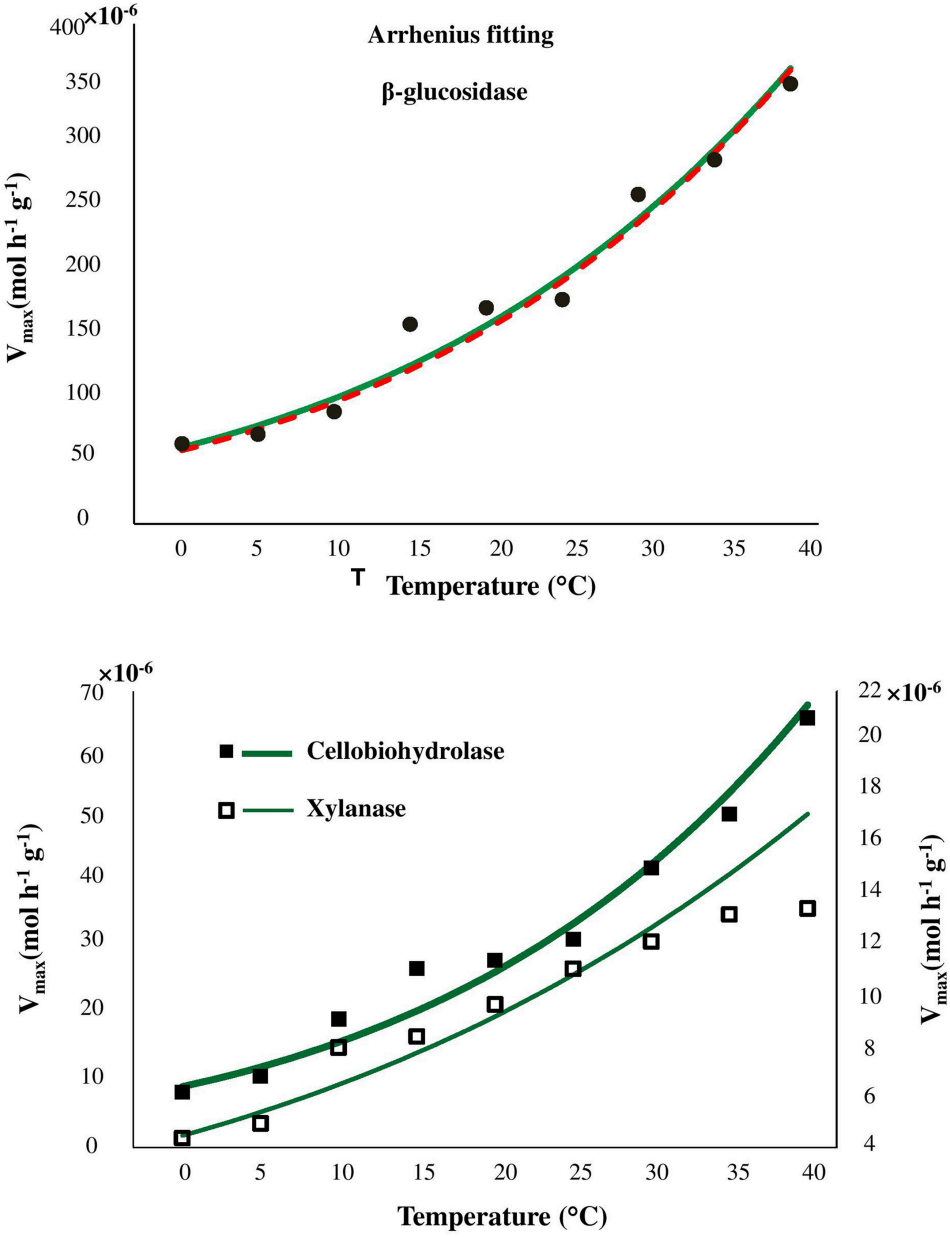
**Fitting of Arrhenius model to experimental ***V***_***max***_ values of β-glucosidase kinetics (top), cellobiohydrolase, and xylanase (bottom), as a function of temperature from 0 to 40°C (green solid line)**. The fitting quality was not improved by application of modified Arrhenius model (red dash line and Table S2).

Temperature sensitivity of all tested enzymes fit well to the Arrhenius equation (Figure 2) with the *E*_*a*_ values ranged from 20.7 to 35.3 kJ mol^−1^ (Table 2). However, the fitting of *V*_*max*_ for all enzymes, especially for β-glucosidase and cellobiohydrolase, to the classical Arrhenius model (Eq. 4) demonstrated deviations within the temperature range 15–30°C (Figure 2, green solid line). The pairwise comparison *t*-test considering temperature increase with 5 degree resolution revealed three groups of stepwise increase of enzyme activities (Figure 1). The accuracy of such a grouping, however, was enzyme-dependent and was less pronounced for enzymes with overall low temperature sensitivity, e.g., xylanase activity differed between lowest and highest temperature (40°C temperature increase) only by a factor of three (Figure 1). Accordingly, the “jumping” pattern was more obvious for β-glucosidase compared to the two other enzymes.

**Table 2:** **The varying ***E***_***a***_-values which were obtained by fitting the Arrhenius equation for three hydrolytic enzymes studied**.

**Enzyme**	**Parameters T (°C)**	**Fitting 0–40**
β-glucosidase	*E_*a*_* (kJ/mol)	31.7±2.57
	A	0.07±0.007
Cellobiohydrolase	*E_*a*_* (kJ/mol)	35.3±2.53
	A	0.06±0.005
Xylanase	*E_*a*_* (kJ/mol)	20.7±2.10
	A	0.045±0.01

*Error values shown are fitting error estimates*.

To prove whether the deviations from Arrhenius function can be explained by enzyme denaturation, we applied the modified Arrhenius equation (5) considering thermal degradation of the enzymes. Fitting to the modified Arrhenius equation (5) demonstrated negligible decreases in catalytic activity, implying an absence of denaturation. Similar *E*_*a*_ values were obtained for the whole temperature range with Equations (5, 6) (Table S2). Furthermore, fitting to Equation (5) did not reduce the deviations of experimental data from the model predictions, suggesting that the revealed grouping (or jumping effect) cannot be explained by thermal degradation of the enzymes. Statistical index *R*^2^ demonstrated similar model efficiency when classical Arrhenius equation were used for fitting as compared with modified Arrhenius equation (Table S2).

**Figure 3:**
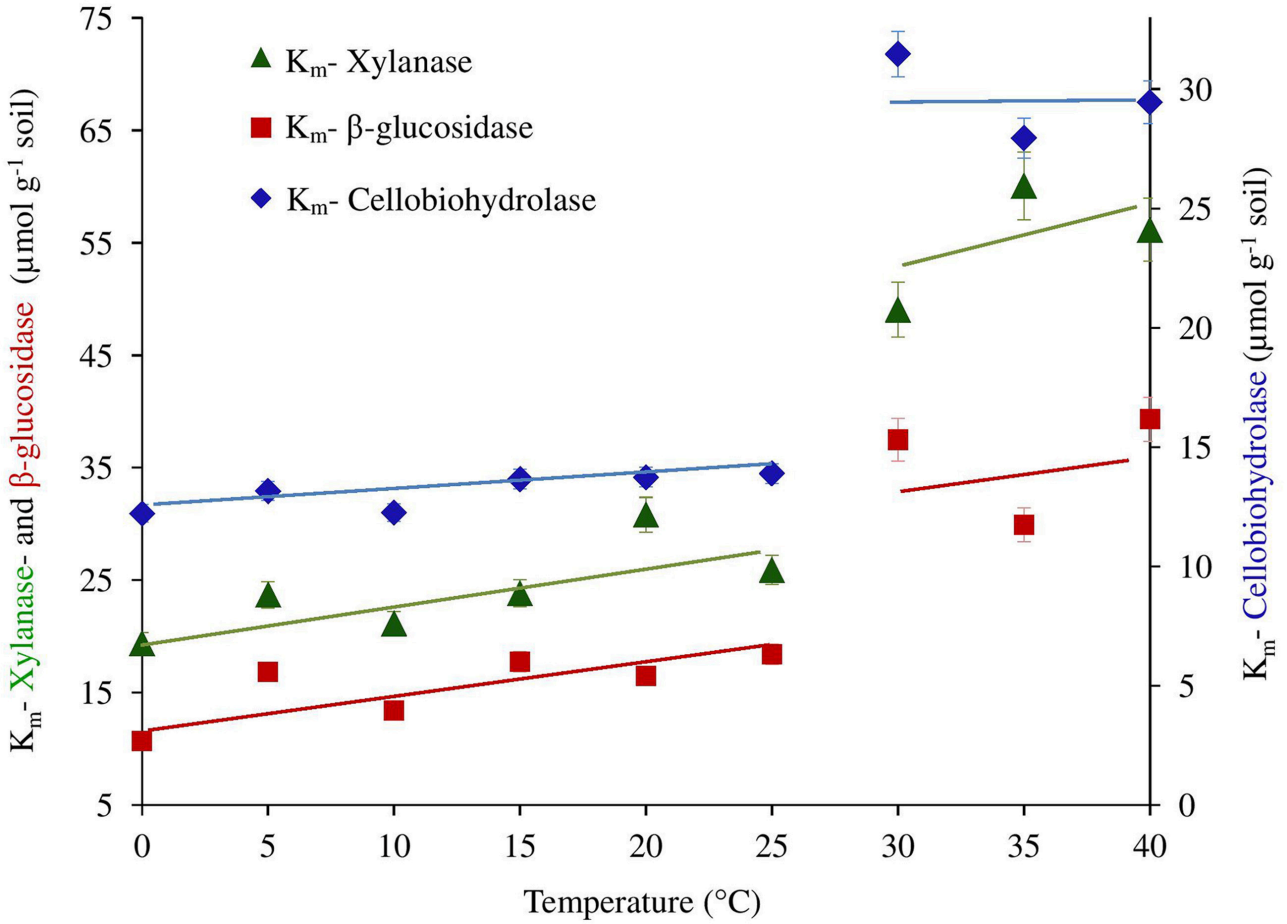
**The ***K***_***m***_ of β-glucosidase, cellobiohydrolase and xylanase plotted as a function of temperature**. Significant differences of *K*_*m*_ values from 25 to 30°C for all enzymes were confirmed by ANOVA (*p* < 0.05), (See Table S1).

We further checked whether the jumping in temperature response of catalytic reactions could be explained by changes in temperature sensitivity of *K*_*m*_. However, the *K*_*m*_ values were relatively constant without significant differences between 0 and 25°C. Thus, the low-temperature “threshold” was due to the sudden 30% increase in *V*_*max*_ over a temperature range of 10–15°C and was unaffected by *K*_*m*_ values (Table 1 and Figure 3). The temperature effect on *K*_*m*_, however, revealed distinct threshold with a significant increase in the affinity of all enzymes to the substrate at temperatures above 30°C (Table 1, Figure 3).

### Canceling effect

**Figure 4:**
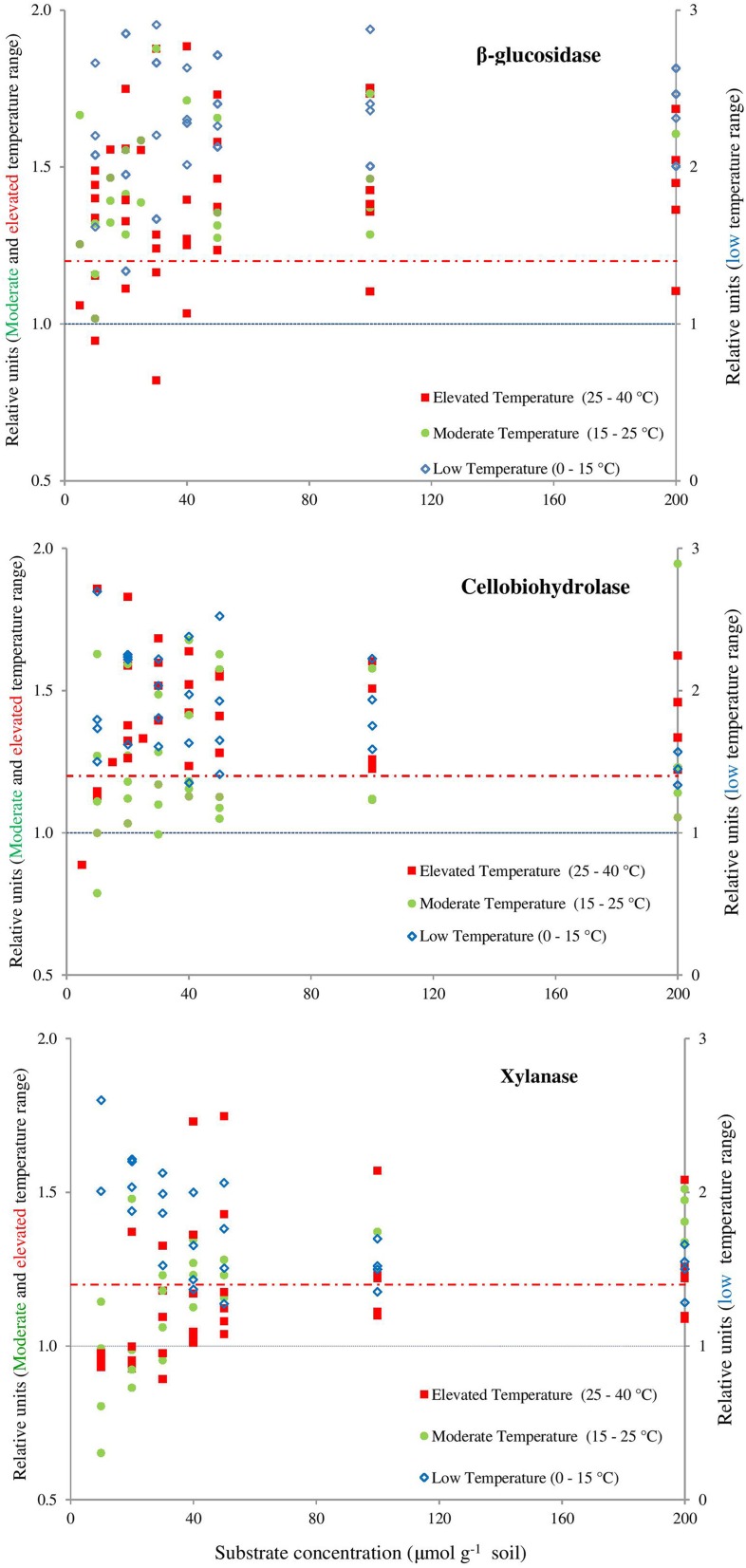
**Presence of the canceling effect in the area below the dashed red line (1.2)**. Symbols represent the values of dividing the reaction rates with 5–10°C resolution for β-glucosidase **(top)**, cellobiohydrolase **(middle)**, xylanase **(bottom)**. Values of *Q*_10_ ≤ 1 indicate full canceling (below blue line) and all values exceeding 1.2, showing the absence of a canceling (e.g., empty blue symbols). Canceling effect is obvious at moderate (15–25°C) and elevated (25–40°C) temperature ranges and mainly at lower substrate concentrations. The area below the dashed red line was arbitrarily accepted as relevant presence of canceling effect if the relative increase of reaction rates is below 20% (means = 1.20 on the Y axis). This threshold considers the uncertainties of the data: the mean SE of each relative unit at the exact concentration of substrate for each enzyme. The error bars were deliberately omitted for clarity.

**Figure 5:**
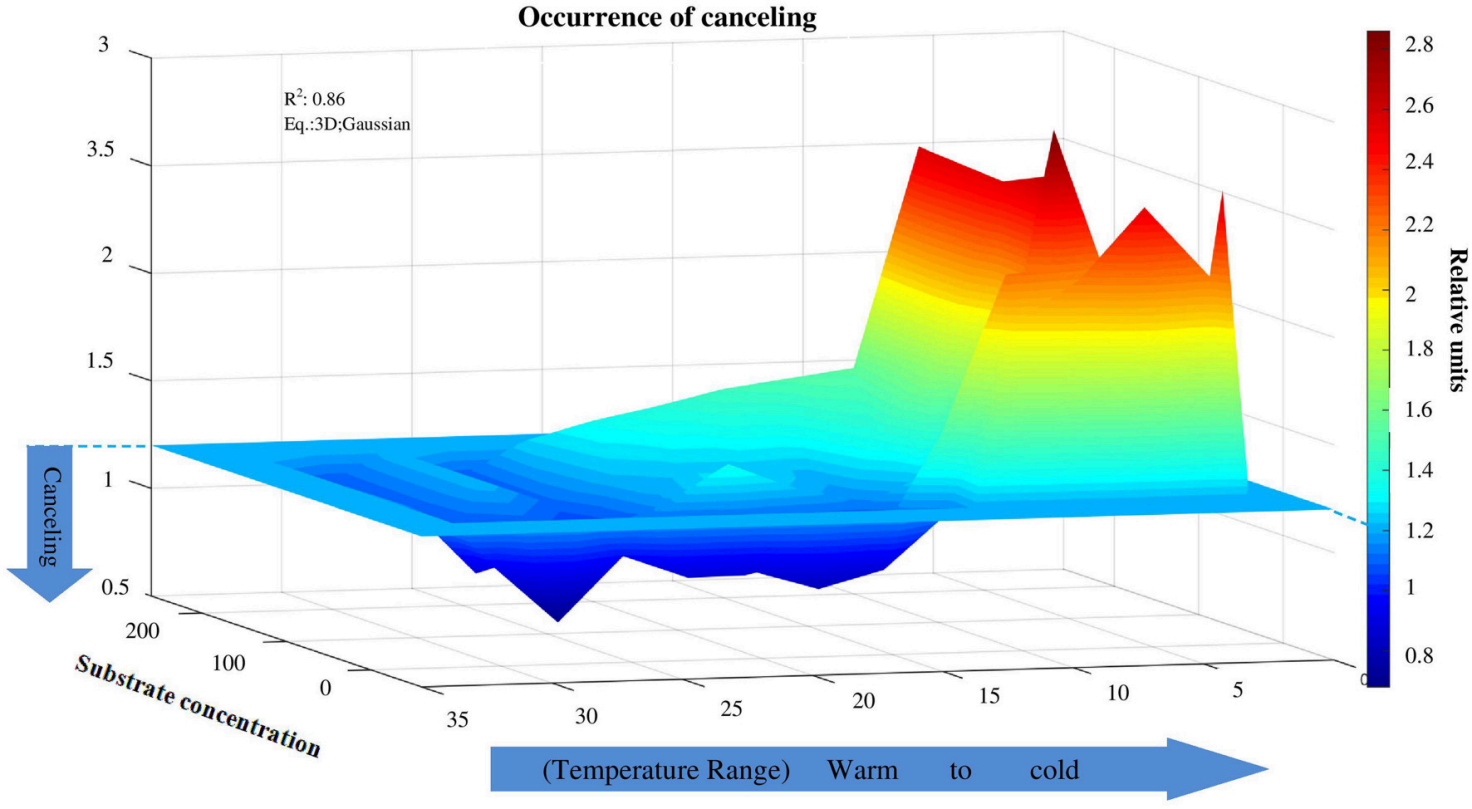
**Canceling effect as a function of temperature and substrate concentration**. Independent of substrate concentration, canceling was never observed at temperatures below 10°C. Canceling effect significantly increased at the intermediate and elevated temperature ranges, especially at low substrate concentration. The canceling effect occurred at warm temperatures at all substrate concentrations for cellobiohydrolase and xylanase.

The canceling effect was pronounced at a temperature increase by 5°C and it was enzyme-specific (Figure 4). Generally, the temperature ranges with observable canceling were wider at substrate concentrations below 50 μmol g^−1^soil (Figures 1, 5). At substrate concentrations above 50 μmol g^−1^soil, canceling was detected within narrow temperature ranges (25–35°C for cellulose-degrading enzymes and 5–25°C for hemicellulolytic enzymes). Independent of substrate concentration, canceling was never observed at temperatures below 10°C or above 35°C (Figure 5).

The canceling effect for 10°C temperature resolution was especially pronounced at low substrate concentration (10–50 μmol g^−1^soil, Figure 5). With one exception for β-glucosidase: at the increase from 15 to 25°C, a canceling effect was detected at the large concentration range of 10–100 μmol g^−1^ soil (Figure 4). Thus, pronounced canceling effects were evident over temperature increments of both 5 and 10°C, but at different thresholds of substrate concentration. With a 10°C increment, the canceling effect was significant only at substrate concentrations below 25 μmol g^−1^ soil. With 5°C resolution, however, the canceling was observed even at saturating substrate concentrations.

## Discussion

### Enzyme responses to temperature

The *Q*_10_ values of the enzyme activities varied from 1.5 to 1.9 within the low temperature range and decreased to 1.4 with increasing temperature. This decrease is in accordance with theoretical predictions (Davidson and Janssens, 2006) and experimental observations of reduced *Q*_10_ values at increased temperature (Xu and Qi, 2001). Our results were in line with common range of *E*_*a*_ for the substrates of three tested enzymes, which varied from 30 to 57 kJ mol^−1^ (Blagodatskaya et al., 2014). Temperature sensitivity of *V*_*max*_ and *K*_*m*_ for the three tested enzymes, with the highest *V*_*max*_**-***Q*_10_ = 2.5 for cellobiohydrolase and the highest *K*_*m*_**-***Q*_10_ = 2.3 for xylanse (Table 1), was in line with studies of German et al. (2012) and Stone et al. (2012). Notably, higher temperature sensitivity of *K*_*m*_ for xylanase suggests that hemicellulose degradation may be more affected by temperature than the other components of plant residues (Stone et al., 2012).

Despite satisfactory approximation of enzyme activities by the Arrhenius equation the fitting demonstrated remarkable deviations within the temperature range 15–30°C. Such deviations were related to the temperature sensitivity of the enzymes and were more pronounced for enzymes with wider range of temperature response (β-glucosidase, cellobiohydolase). Deviations of experimental data from both classic and modified Arrhenius models have been observed previously for parameters of microbial activity, e.g., for microbial respiration and growth (Ratkowsky et al., 1983; Pietikäinen et al., 2005; Sand-Jensen et al., 2007). Furthermore, an inconsistent response of enzyme activity to gradual temperature increase was also observed (but not discussed) for β–glucosidase in soils of Alaska and Costa Rica (Figure 1 in German et al., 2012). Such deviations from Arrhenius equation have been partly explained by protein denaturation at temperatures above 35°C (Daniel et al., 1996; Goyal et al., 2014) or by experimental artifacts arising from changes of substrate pools over time that are more significant at higher temperatures (Sand-Jensen et al., 2007). In our study, however, the fitting quality was not improved when denaturation was considered by the modified Arrhenius model, suggesting a negligible effect of thermal denaturation in the temperature range 0–40°C (Goyal et al., 2014). Besides that, enzymes immobilization by clays or entrapment by organic matter (Allison, 2006; Burns et al., 2013) indirectly alter enzymes kinetics (Nannipieri and Gianfreda, 1998; Nannipieri et al., 2011) and increase stability against thermal denaturation and proteolysis (Burns, 1982; Stotzky, 1986; Sarkar et al., 1989; Nannipieri et al., 1996; Nielsen et al., 2006; Maire et al., 2013). Since, thermal denaturation of enzymes cannot explain the deviations from Arrhenius function in our study, other factors, such as, enzyme immobilization, changes in microbial community structure or shifts in community function (resulting in expression of iso-enzymes) altering temperature sensitivity of the enzyme kinetic parameters (*V*_*max*_ and *K*_*m*_), must be responsible for this behavior (Davidson and Janssens, 2006).

The parameters of Michaelis-Menten kinetics responded differently to temperature increase at low and high temperature thresholds because the sensitivities of *V*_*max*_ and *K*_*m*_ do not always correspond to each other (Stone et al., 2012; Tischer et al., 2015). The *V*_*max*_ values raised by 30–100% at the temperature threshold between 10 and 15°C. Such an increase in *V*_*max*_ was not accompanied by significant changes in the *K*_*m*_ values. Thus, the enzyme activity (*V*_*max*_) strongly increased, but the enzyme systems (*K*_*m*_) themselves remained unaffected. The absence of significant trends in enzyme affinities (*K*_*m*_) within the range of 0–25°C shows that microorganisms in the studied Luvisol at temperate climate are adapted to diurnal temperature changes and produce a similar enzyme sets below 25°C. The deviations from Arrhenius behavior at 10–15°C therefore cannot be explained by the temperature sensitivity of *K*_*m*_. Thus, the raised enzyme activity between 10 and 15°C, could indicate the increase in enzyme production due to the shift in domination of mesophilic microbial group with optimal growth temperature at 15–25°C. Such microorganisms can occur and produce similar extracellular enzymes at both psychrophilic and mesophilic temperature ranges. However, their activity and enzymes production increases under optimal temperature resulting in local non-linear temperature response. This was supported by the absence of a canceling effect at low temperature (0–10°C) and by its occurrence at temperatures above 15°C indicating weaker temperature sensitivity of mesophilic microorganisms. A two-fold increase of *K*_*m*_ between 25 and 30°C indicated strong reduction of enzyme affinities to the substrate due to production of iso-enzymes (Hochachka and Somero, 2002). Thus, “jumping” temperature sensitivity of soil enzymes at 30°C is related to the shift toward temperature-dependent physiological groups producing enzyme isoforms with similar functions, but different temperature sensitivities (Khalili et al., 2011; Bradford, 2013). The uncommon high temperatures affect not only the active microorganisms, but also activate dormant microorganisms (Birgander et al., 2013) expressing the enzymes beneficial in thermophilic range (Hochachka and Somero, 2002; Pepper et al., 2011; Yumoto, 2013). Therefore, the major shift in the temperature response of soil microbial activity above 30°C was expected, because these temperatures are uncommon under a temperate climate with annual soil temperature of 8–10°C (Bárcenas-Moreno et al., 2009).

The observed temperature thresholds with strong temperature sensitivity were clearly obvious for β–glucosidase, the enzyme responsible for decomposition of cellulose. These thresholds were also observable for other enzymes involved in C cycle responsible for the decomposition of cellulose and hemicelluloses. However, we expect that the thresholds, canceling effects and mechanisms of sensitivity may be different for the enzymes of other cycles: N, P, S, as well as the broad group of oxidative enzymes, like peroxidases and phenoloxidases. Furthermore, to generalize the conclusions based on one soil type from temperate climate, more soils from various climate zones need to be tested. Therefore, we need more mechanistic work, *in situ* studies along with the studies of pure and isolated enzymes from a range of habitats to verify the findings on temperature sensitivity of hydrolytic reactions and to relate them with functional and phylogenic structure of microbial community as well as with temperature responses of specific proteins.

### Canceling effect

**Figure 6:**
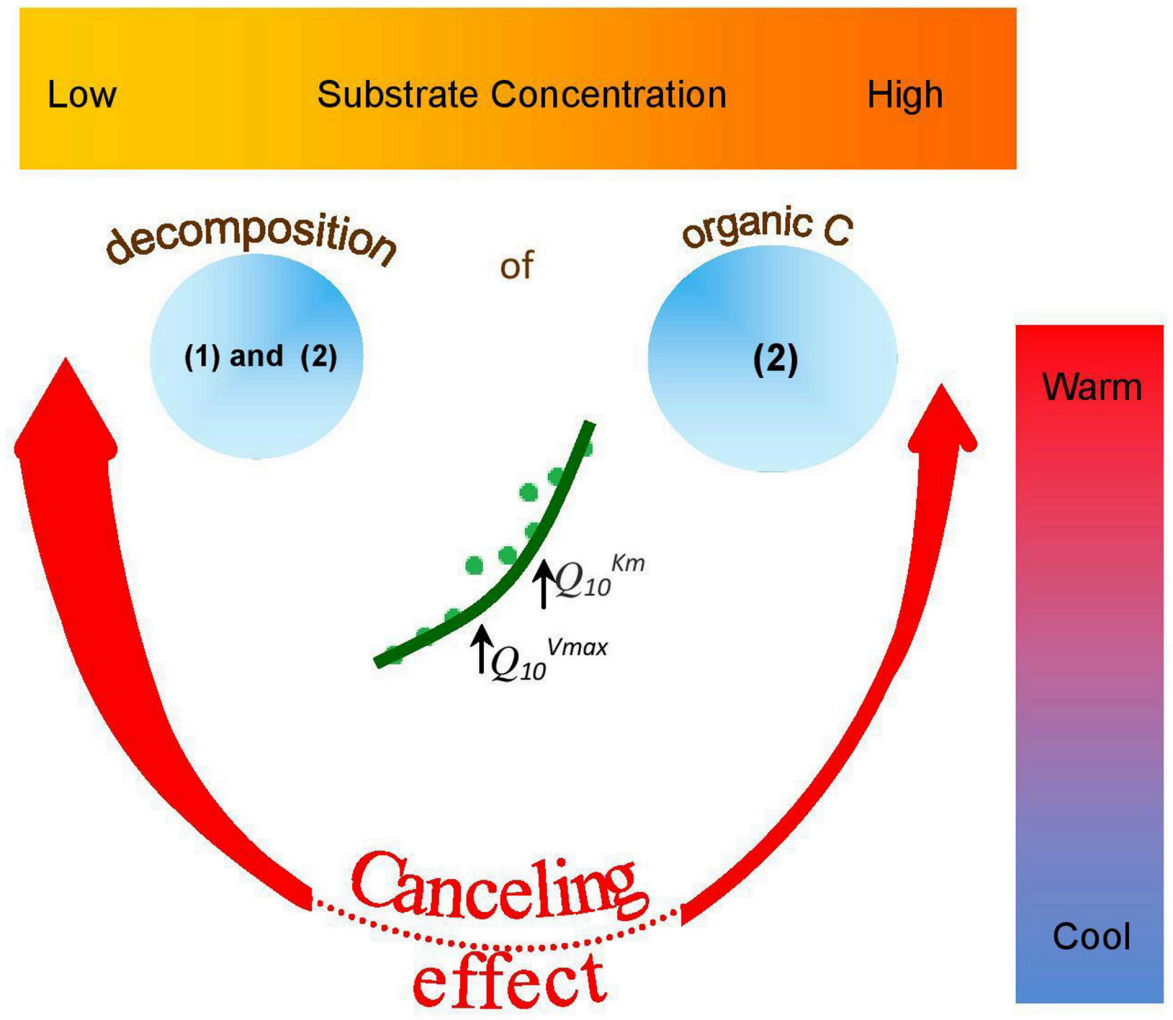
**Occurrence and potential outcome of “a canceling effect” in soil of temperate ecosystem as affected by temperature and substrate concentration**. Canceling effect is more pronounced at warm temperatures (red arrows) and at low (thick arrow) than at high (thin arrow) substrate concentration. Canceling does not occur at low temperatures (dotted red line). Canceling occurrence at moderate temperatures was accompanied by deviations of reaction rates (green circles) from Arrhenius function (green line). This was mainly due to strong increase of *Q*^*Vmax*^_10_. Next deviation from Arrhenius function at warm temperatures was mainly explained by increase of *Q*^*Km*^_10_. As a result of canceling, the decomposition of: (1) recalcitrant C (Ågren and Wetterstedt, 2007; Larionova et al., 2007; Gershenson et al., 2009) and (2) cellulose and hemicellulose (based on our study) will be slowed down. Therefore, at low substrate concentrations and warm temperatures, canceling effect can be considered as natural mechanism mitigating accelerated SOM decomposition as a consequence of global warming.

The canceling effect was not significant at lower temperatures (0–10°C), but its significance increased at 15–25°C for all enzymes tested (Figure 6). This finding supports the theoretical predictions that *V*_*max*_ and *K*_*m*_ can cancel each other upon temperature increase (Davidson et al., 2006) especially at the intermediate and elevated temperatures that favors mesophilic microorganisms. Thus, reduced temperature sensitivity of hydrolytic reactions and a smoothed response to warming could be predicted within the mesophilic temperature range for similar soils under temperate climate. Canceling occurrence at moderate temperatures was accompanied by deviations of reaction rates from Arrhenius function (Figure 6). This was mainly due to strong increase of *Q*_10−_*V*_*max*_. Next deviation from Arrhenius function at 25–30°C was mainly explained by increase of *Q*_10−_*K*_*m*_. The occurrence of a canceling effect for cellobiohydrolase and xylanase at warm temperatures (30–35°C) at all substrate concentrations suggested slower decomposition of the main components of plant residues (cellulose and hemicelluloses) than predicted by the Arrhenius equation, even at increased net primary production (Figure 6). As a result of canceling, the decomposition of recalcitrant C (Ågren and Wetterstedt, 2007; Larionova et al., 2007; Gershenson et al., 2009) and specifically of cellulose and hemicellulose (based on our study) will be slowed down. The canceling therefore can be considered a natural mechanism reducing the consequences of global warming for microbial decomposition of soil organics predicted in temperate ecosystems (Tang and Riley, 2014). Under cold climate, however, the regulation of hydrolytic activity by canceling in response to warming could be of minor relevance given that canceling was never observed at temperatures below 10°C (Figure 5). As this conclusion has been done for soil from temperate ecosystem, the relevance of canceling mechanism responsible for weaker soil carbon-climate feed-backs needs to be proven for the soils with contrasting properties (e.g., texture, structure, pH, C content, etc.) in the range of climate zones, e.g., in boreal and tropical environments. Consideration of sensitivity jumping and temperature thresholds can significantly improve the predictions of Earth system models for C cycling at regional and global levels, especially if the response of broad range of functional enzymes is evaluated. To our knowledge, this is the first study explored the canceling effect across a complete range of substrate concentrations in soil for the set of hydrolytic enzymes at a temperature resolution of 5°C increments (nine temperature levels). This study confirms previous studies on “apparent” *K*_*m*_ (Tabatabai, 1994; Nannipieri and Gianfreda, 1998; Marx et al., 2005) and provides a basis for implementing both *V*_*max*_ and *K*_*m*_ in models of soil organic matter decomposition under global warming (German et al., 2012). In particular, because *K*_*m*_ and *V*_*max*_ vary independently as temperature changes (Stone et al., 2012), both parameters of the Michaelis-Menten kinetics need to be considered in global earth system models, especially at temperature thresholds for physiological groups of soil microorganisms.

### Conflict of interest statement

The authors declare that the research was conducted in the absence of any commercial or financial relationships that could be construed as a potential conflict of interest.

## Supplementary material

Data supporting Figures 1, 2 is available as in Supporting Information Tables S1, S2.
