# Supplementary material for: Nonlinear temperature sensitivity of enzyme kinetics explains canceling effect—a case study on loamy haplic Luvisol
Source: Front Microbiol. 2015 Oct 14;6:1126. doi: 10.3389/fmicb.2015.01126 (PMC4604301; doi:10.3389/fmicb.2015.01126)
Supplement: Supplementary file 1 [file DataSheet1.DOC]

Supplementary Material

**Nonlinear temperature sensitivity of enzyme kinetics explains canceling effect- a case study on** **loamy haplic Luvisol**

Bahar S. Razavi, Evgenia Blagodatskaya, Yakov Kuzyakov

*** Correspondence:** Corresponding Author: [janeblag@mail.ru](mailto:janeblag@mail.ru)

# Supplementary Tables

Analysis of variance (ANOVA) was used to test the significant differences of K_m_ values from 25 to 30 °C for all enzymes (p<0.05). Fitting by modified Arrhenius equation demonstrated negligible decrease in catalytic activity when denaturation was considered. Similar *E_a_* values were obtained for the whole range of temperatures by Eq. 5 and 6

Table S1. The significant differences of *K_m_* values from 25 to 30 °C for all enzymes were tested with ANOVA (p<0.05).

| Enzyme | Temperature-range groups (°C) | *p* value of *K_m_* |  |
| --- | --- | --- | --- |
|  |  |  |  |
| Xylanase  Cellobiohydrolase  β-glucosidase | 25-30 | 0.004 |  |
|  | 25-30 | 0.005 |  |
|  |  |  |  |
|  | 25-30 | 0.0072 |  |
|  |  |  |  |

**Table S2**. Fitting by modified Arrhenius equation demonstrated negligible decrease in catalytic activity when denaturation was considered. Similar *E_a_* values were obtained for the whole range of temperatures by Eq. 5 and 6

| **β–glucosidase** | | | **Cellobiohydrolase** | **Xylanase** |
| --- | --- | --- | --- | --- |
| **Temperature (°C)** | ***V_max_* for whole range of temperatures** | **Denaturation rate for all steps** | ***V_max_* for whole range of temperatures** | ***V_max_* for whole range of temperatures** |
| 0 | 0.000000061 | 0.000000059 | 0.000000010 | 0.0000000048 |
| 1 | 0.000000064 | 0.000000062 | 0.000000011 | 0.0000000049 |
| 2 | 0.000000068 | 0.000000065 | 0.000000011 | 0.0000000051 |
| 3 | 0.000000071 | 0.000000069 | 0.000000012 | 0.0000000053 |
| 4 | 0.000000075 | 0.000000072 | 0.000000013 | 0.0000000054 |
| 5 | 0.000000079 | 0.000000076 | 0.000000013 | 0.0000000056 |
| 6 | 0.000000083 | 0.000000080 | 0.000000014 | 0.0000000058 |
| 7 | 0.000000087 | 0.000000084 | 0.000000015 | 0.0000000060 |
| 8 | 0.000000091 | 0.000000088 | 0.000000016 | 0.0000000062 |
| 9 | 0.000000096 | 0.000000093 | 0.000000016 | 0.0000000064 |
| 10 | 0.000000100 | 0.000000097 | 0.000000017 | 0.0000000066 |
| 11 | 0.000000105 | 0.000000102 | 0.000000018 | 0.0000000068 |
| 12 | 0.000000110 | 0.000000107 | 0.000000019 | 0.0000000070 |
| 13 | 0.000000115 | 0.000000112 | 0.000000020 | 0.0000000072 |
| 14 | 0.000000121 | 0.000000118 | 0.000000021 | 0.0000000074 |
| 15 | 0.000000127 | 0.000000123 | 0.000000022 | 0.0000000077 |
| 16 | 0.000000133 | 0.000000129 | 0.000000024 | 0.0000000079 |
| 17 | 0.000000139 | 0.000000135 | 0.000000025 | 0.0000000081 |
| 18 | 0.000000145 | 0.000000142 | 0.000000026 | 0.0000000084 |
| 19 | 0.000000152 | 0.000000148 | 0.000000027 | 0.0000000086 |
| 20 | 0.000000159 | 0.000000155 | 0.000000029 | 0.0000000089 |
| 21 | 0.000000166 | 0.000000162 | 0.000000030 | 0.0000000091 |
| 22 | 0.000000173 | 0.000000170 | 0.000000032 | 0.0000000094 |
| 23 | 0.000000181 | 0.000000178 | 0.000000033 | 0.0000000097 |
| 24 | 0.000000189 | 0.000000186 | 0.000000035 | 0.0000000100 |
| 25 | 0.000000197 | 0.000000194 | 0.000000037 | 0.0000000102 |
| 26 | 0.000000206 | 0.000000203 | 0.000000039 | 0.0000000105 |
| 27 | 0.000000215 | 0.000000212 | 0.000000040 | 0.0000000108 |
| 28 | 0.000000224 | 0.000000221 | 0.000000042 | 0.0000000111 |
| 29 | 0.000000234 | 0.000000231 | 0.000000044 | 0.0000000114 |
| 30 | 0.000000244 | 0.000000241 | 0.000000047 | 0.0000000118 |
| 31 | 0.000000254 | 0.000000251 | 0.000000049 | 0.0000000121 |
| 32 | 0.000000265 | 0.000000262 | 0.000000051 | 0.0000000124 |
| 33 | 0.000000276 | 0.000000273 | 0.000000053 | 0.0000000127 |
| 34 | 0.000000287 | 0.000000285 | 0.000000056 | 0.0000000131 |
| 35 | 0.000000299 | 0.000000297 | 0.000000058 | 0.0000000134 |
| 36 | 0.000000311 | 0.000000309 | 0.000000061 | 0.0000000138 |
| 37 | 0.000000324 | 0.000000322 | 0.000000064 | 0.0000000142 |
| 38 | 0.000000337 | 0.000000335 | 0.000000067 | 0.0000000145 |
| 39 | 0.000000350 | 0.000000349 | 0.000000070 | 0.0000000149 |
| 40 | 0.000000364 | 0.000000363 | 0.000000073 | 0.0000000153 |
